# Comparison of visceral fat lipolysis adaptation to high-intensity interval training in obesity-prone and obesity-resistant rats

**DOI:** 10.1186/s13098-022-00834-9

**Published:** 2022-05-03

**Authors:** Yang Liu, Yu Li, Baishuo Cheng, Shige Feng, Xiangui Zhu, Wei Chen, Haifeng Zhang

**Affiliations:** 1 Physical Education College, Hebei Normal University, Shijiazhuang, China; 2 Hebei Provincial Key Laboratory of Measurement and Evaluation in Human Movement and Bio-Information, Hebei Normal University, Shijiazhuang, China

**Keywords:** HIIT, Obesity prone, Obesity resistance, Visceral adipose, Adrenergic receptors

## Abstract

**Background/objectives:**

Visceral obesity is one of the key features of metabolic syndrome. High-intensity interval training (HIIT) could effectively reduce visceral fat, but its effects show strong heterogeneity in populations with different degrees of obesity. The mechanism may be related to the differential adaptation to training between obesity phenotypes, namely obesity prone (OP) and obesity resistant (OR). The aim of the present study was to compare adaptive changes of visceral adipose lipolysis adaptation to HIIT between OP and OR animals and further explore the upstream pathway.

**Methods:**

OP and OR Sprague Dawley rats were established after feeding a high-fat diet for 6 weeks; they were then divided into HIIT (H-OP and H-OR) and control (C-OP and C-OR) groups. After 12 weeks of HIIT or a sedentary lifestyle, animals were fasted for 12 h and then sacrificed for histology as well as gene and protein analysis. Visceral adipocytes were isolated without fasting for catecholamine stimulation and β3-adrenergic receptor (β3-AR) blockade in vitro to evaluate the role of upstream pathways.

**Results:**

After training, there were no differences in weight loss or food intake between OP and OR rats (P > 0.05). However, the visceral fat mass, adipocyte volume, serum triglycerides and liver lipids of OP rats decreased by more than those of OR rats (P < 0.05). Meanwhile, the cell lipolytic capacity and the increase in the expression of β3-AR were higher in the OP compared with OR groups (P < 0.05). Although training did not increase sympathetic nervous system activity (P > 0.05), the cell sensitivity to catecholamine increased significantly in the OP compared with OR groups (P < 0.05). Following blocking β3-AR, the increased sensitivity disappeared.

**Conclusion:**

With HIIT, OP rats lost more visceral fat than OR rats, which was related to stronger adaptive changes in lipolysis. Increased β3-AR expression mediated this adaptation.

## Introduction

Because of the more pronounced association of excessive visceral adipose tissue (VAT) accumulation with metabolic syndrome, a loss of VAT may provide more health benefits than total fat. High-intensity interval training (HIIT) is a type of protocol that alternates high-intensity training periods with low-intensity intervals. With the active or passive intervals, HIIT can enable participants to complete high-intensity exercise with a higher training volume. Over the past decade, HIIT has become a hot topic of health promotion: compared with classic aerobic training, it has shown similar or better effects on visceral and liver fat loss, improving insulin resistance (IR) and cardiovascular function while being more time-efficient [1–4].

Numerous studies have shown that HIIT can effectively reduce visceral fat [1, 5–7]; however, other experiments have found no change in VAT mass after training [5, 8–13]. Moreover, even in studies confirming that HIIT reduces VAT, individual differences in the reduction are huge (standard deviations up to ten times larger than means) among different subjects with the same protocol [1]. Different responses to the same intervention indicate that individual differences can strongly affect the VAT-reducing effect of HIIT. In their meta-analysis, Maillard et al. [5] confirmed that HIIT significantly reduces visceral fat in subjects with overweight and obesity but has no effect on normal weight people, suggesting that individual differences in the obesity degree and lipid metabolism may be the key factors affecting the VAT reducing effect of HIIT.

Two metabolic phenotypes, obesity prone (OP) and obesity resistant (OR), exist due to genetic polymorphisms. OP individuals are more likely to accumulate fat than OR individuals when exposed to obesity-causing factors (diet, sedentary lifestyle etc.) [14, 15]. The causes of OP and OR are related to differences in how feeding behaviour, physical activity (PA) and the sympathetic nervous system (SNS) regulate adipose catabolism. With the same high-fat diet (HFD), compared with OR subjects, OP subjects tend to show higher food intake and lower PA [16, 17], lower secretion of norepinephrine (NE, the main central messenger promoting lipolysis) [18], a higher respiratory quotient (lower lipid oxidation) [19] and a tendency for triglycerides (TG) to accumulate in adipose tissue rather than muscle [18]. The exercise responses and adaptations of OP and OR subjects are also different. While the mechanisms by which food intake and PA change are still debated, after the same aerobic training, OP subjects seem to show greater leptin reduction and improvement in IR compared with OR subjects [20–22]. However, few studies have been published that compare the differential adaptation to HIIT between OP and OR individuals, especially in term of VAT metabolism.

Because the main energy substrate during exercise is glycogen, the mechanism by which HIIT can reduce fat is generally suspected to be related to the ‘post-exercise’ changes in adipose catabolism [23, 24]. Catecholamine release by the SNS and adrenal glands can activate lipolysis in adipocytes through β3-adrenergic receptors (β3-AR), which are the major regulation pathway of adipose catabolism [25]. In previous studies, we and other teams found that long-term HIIT could cause several adaptive changes in this pathway. HIIT increases the levels of β3-AR messenger RNA (mRNA) [26] and hormone-sensitive lipase (HSL, a catecholamine-regulated key lipolytic enzyme) [27] after NE stimulation in vitro. Moreover, the visceral adipocytes of mice subjected to HIIT show stronger HSL phosphorylation and TG hydrolysis [28], which means that VAT could be mobilised more easily during hunger, cold or stress. However, whether HIIT has different effects on OP and OR individuals remains unclear. A recent large-sample survey found that increased energy consumption during exercise would lead to a decrease in the basal metabolic rate during the recovery period. Furthermore, this ‘compensation’ phenomenon is stronger in subjects with obesity than in normal-weight people [29], which means that people with obesity would have greater difficulty losing fat by exercising. If HIIT could provide greater post-exercise catabolism in OP compared with OR individuals, it should be recommended as an efficient protocol that better alleviates the ‘energy compensation’ for the OP population.

Based on the above considerations, we compared the fat loss effect and influence of HIIT on VAT lipolysis between OP and OR rats. First, Sprague Dawley rats were fed an HFD for 6 weeks to establish an OP/OR animal model, and then subjected to 12 weeks of HIIT. After sacrificing the rats, we examined the morphology of adipocytes, hepatocytes and mitochondria, the mRNA expression of TBX1 (a marker of thermogenesis) and the protein expression of tyrosine hydroxylase (TH, a marker of SNS transmitter release), β3-AR, HSL, and HSL phosphorylated at serine 660 (HSLser660, a marker of HSL activation). We found that β3-AR expression rather than SNS regulation might play a key role in increased VAT catabolism in OP rats. Therefore, we performed an in vitro experiment using catecholamine stimulation and β3-AR blockade. The findings confirmed that the greater fat lipolysis of OP rats was related to increased β3-AR expression.

## Materials and methods

### Study design

**Fig. 1:**
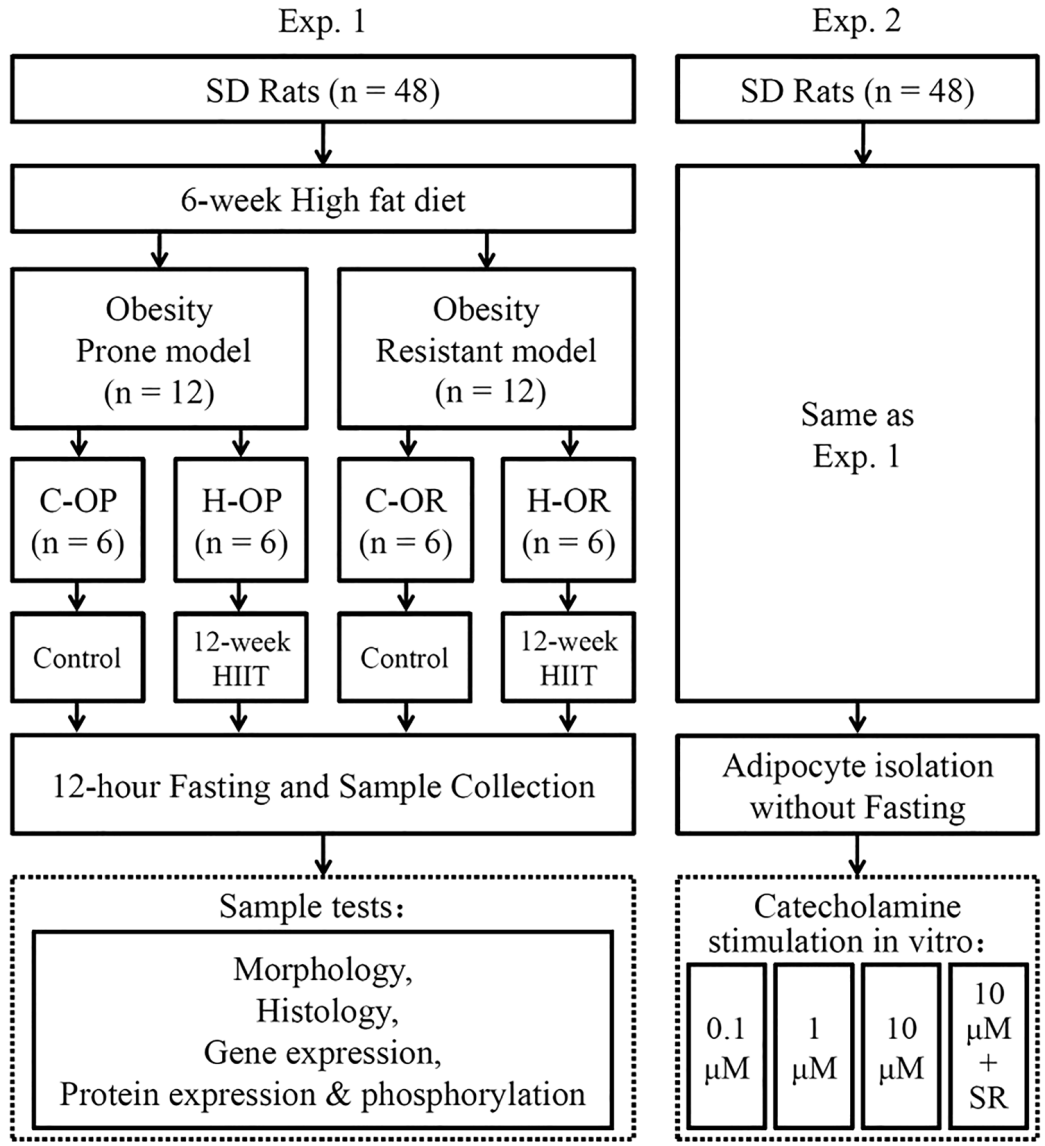
Study design. *C-* the control groups; *H-* the groups subjected to high-intensity interval training; *OP* obesity prone; *OR* obesity resistant; *0.1, 1 and 10 μM* the gradient of isoproterenol stimulation; *SR* SR59230a, a selective antagonist of β3-adrenergic receptors

We performed two parallel experiments: experiment 1 (Exp. 1) to compare fasting-induced lipolysis in vivo and experiment 2 (Exp. 2) to examine the receptor sensitivity to catecholamine in vitro after excluding neural control (Fig. 1). In Exp. 1, after feeding Sprague Dawley rats an HFD for 6 weeks, the OP (1st quartile of body weights, n = 12) and OR (4th quartile of body weights n = 12) rats were separated into –OP and –OR groups and further randomly divided into control (C-) and training (H-) groups. After 12 weeks of HIIT or a sedentary lifestyle, the rats were rested for 48 h, fasted for 12 h and then sacrificed for analysis. In Exp. 2, the rats received the same feeding and exercise intervention, but they did not fasted before sacrifice. Then, visceral adipocytes were isolated for in vitro stimulation and receptor blocking.

### Establishment of the OP/OR rat model

The OP/OR model was established according to classic methods [14, 15]. Forty-eight specific-pathogen free male Sprague Dawley rats (7 weeks old; Changsheng Biotech) were housed under controlled conditions (24 ± 2 °C, humidity of 50% ± 5%, 12-h photoperiod). After 1 week of feeding with an adaptive standard chow, the rats were fed an HFD for 6 weeks; it comprised 60% standard chow, 16% sugar, 5% fat, 18% egg yolk powder and 1% sodium cholate. Subsequently, the rats were sorted by body weight. The first quartile (n = 12) of rats was selected as the OP model and the fourth quartile (n = 12) was selected as the OR model. HFD feeding continued throughout the next training period.

### Exercise protocol

After 3 days of adaptive training (10 min/day, 5 m/min, 0° incline), the exercise groups were subjected to 12 weeks of 25° uphill treadmill HIIT with 5 days of training and 2 days of rest every week. Each HIIT contained multiple 1 min high running speed–1 min medium running speed cycles. Over 12 weeks, the medium running speed gradually increased from 12 to 20 m/min, and the high speed gradually increased from 22 to 34 m/min, which was adjusted based on an incremental exercise test (IET). The distance of each HIIT was equal to 45 min of running at medium speed. See our previous study for more details [28].

### Sample collection

After 48 h of rest, rats of Exp. 1 were fasted for 12 h and then sacrificed after anaesthesia (intraperitoneal pentobarbital sodium, Solarbio, China), while rats of Exp. 2 were sacrificed without fasting. The epididymal fat pads were collected to examine VAT. After weighing, the central part of the fat pads were removed and fixed in 4% paraformaldehyde or 4% glutaraldehyde for optical microscopy or transmission electron microscopy (TEM). The remaining tissues were frozen at -80 °C. Fat pads of rats in Exp. 2 were minced and put into tubes containing phosphate-buffered saline for adipocyte isolation; they were not frozen.

### Histological assessment of adipocyte volume, liver lipids and mitochondria

The adipocyte volume was assessed by haematoxylin and eosin (H&E) staining. Briefly, after fixation in 4% paraformaldehyde for 4 h, tissue was embedded in paraffin, cut into 5 μm sections, dewaxed, rehydrated, stained with haematoxylin (5 min), washed with water and 0.6% ammonia and finally stained with eosin (2 min). After dehydration and sealing, the tissue was observed under an optical microscope. Two hundred adipocytes per group were randomly selected for cell areas analysis using ImageJ 1.51.

Liver fat deposition was analysed with Oil red O staining. Briefly, fixed liver was stained with Oil red O (9 min), equilibrated in 60% isopropanol, stained with haematoxylin (3 min), equilibrated in 60% alcohol, washed in tap water and then observed under a microscope. The grey value of the red signal was analysed with ImageJ 1.51.

To observe the number and distribution of mitochondria, after fixation in glutaraldehyde, the tissue was washed with 0.07 M phosphate buffer, fixed with 1% osmic acid (2 h), dehydrated with an acetone gradient at 4 °C, infiltrated and sealed with epoxy resin at 37 °C and then allowed to stand at 60 °C for 48 h. Subsequently, the tissue was sliced into 70 nm ultrathin sections, dyed with uranyl acetate and lead citrate and photographed by using a transmission electron microscope (HITACHI H-7650, Japan).

### Blood lipids

All samples were analysed using established enzymatic assays kits (Jiancheng Bio, China) for total cholesterol (TC), triglycerides (TG), low-density lipoprotein Cholesterol (LDL-C) and high-density lipoprotein cholesterol (HDL-C). Six randomly selected samples were measured twice and all intraclass correlation coefficients (ICC) were > 0.75 (good reliability).

### Protein and RNA expression

The protein expression of HSL, phosphorylated HSL-ser660, TH and β3-AR was examined by western blot. Briefly, the tissue was chopped and homogenised with radioimmunoprecipitation assay (RIPA) buffer (Solarbio, China) with phosphatase inhibitor (Thermo Fisher, USA) diluted 1:100, centrifuged at 12,000 rpm for 20 min at 4 ℃ and then boiled at 98 °C for 5 min. The samples were subjected to sodium dodecyl sulphate–polyacrylamide gel electrophoresis using 10% gels. The separated protein was then transferred onto polyvinylidene fluoride membranes. The membranes were incubated with the following primary antibodies: anti-HSL (1:2000; Cell Signaling Technology), anti-HSL-ser660 (1:2000; Cell Signaling Technology, USA), anti-TH (1:2000; Cell Signaling Technology, USA), and anti-β3-AR (1:1000; Abcam, USA). Actin was used as loading control (1:5000; Bioworld, China). After incubation with the secondary antibody (1:5000; Solarbio, China), luminescence was analysed by using a gel imaging system (Fusion Fx5, VILBER LOURMAT, France).

TBX1 mRNA expression was examined by using quantitative real-time polymerase chain reaction (PCR). Briefly, tissue was homogenised with TRIzol reagent (Thermo Fisher) (1:10 ratio). Total RNA was separated after extraction and precipitation. A portion of the isolated RNA was reverse transcribed using a complementary DNA (cDNA) transcription kit (Servicebio, China). The reactions were run on a Bio-Rad real-time PCR system (USA) and the relative expression of TBX1 was calculated by using the 2^−∆∆CT^ method, in which ∆∆CT = [(CT_TBX1_ − CT_β-actin_)_treatment_] − [(CT_TBX1_ − CT_β-actin_)_nontreatment_].

### Primary adipocyte isolation and catecholamine-induced lipolysis in vitro

Primary visceral adipocytes were isolated from tissue after incubation with collagenase (Solarbio, China) for 30 min at 37 °C. The isolated adipocytes were stimulated with an isoproterenol (ISO) gradient (0.1, 1 and 10 μM, Sigma-Aldrich, USA). To determine whether ISO-induced lipolysis is activated through β3-AR, SR59230a (MedChemExpress, USA, 100 nM), a selective antagonist, was added alongside 10 µM ISO. The methods of isolation and in vitro stimulation have been published previously [28, 30]. The amount of glycerol released by cells—a marker of lipolysis—was measured by using an enzyme labelling kit (Jiancheng Bio, China). For quality control, twenty randomly selected samples were measured twice and the ICC was 0.814 (> 0.75 indicates good reliability).

### Statistical analysis

The data are presented as the mean ± standard deviation (SD). Body weight changes over time among the groups (OP/OR, HIIT/sedentary) were analysed by using repeated-measures analysis of variance (ANOVA), with time and group as the main factors as well as the time × group cross-effect. Food intake during the model establishment period and cell glycerol release with or without β3-AR blockade were analysed by using independent samples t-test. After 12 weeks of training, the influences of HIIT between OP and OR rats regarding body weight, food intake during training, fat weight, adipocyte area, blood and liver lipids, protein/gene expression and glycerol release were compared with 2 × 2 two-way ANOVA (cross-effect: HIIT/sedentary × OP/OR). When the cross-effect was significant, simple effect analysis was used to compare the differences between groups. Due to the non-normal distribution, TBX1 expression was analysed by using the Kruskal–Wallis test. Statistical significance was set at P < 0.05. Based on the fat pad mass data from previous experiments, the minimum sample size was 4 (α = 0.05, power = 0.8).

## Results

### Body weight and food intake during the model establishment period

**Fig. 2:**
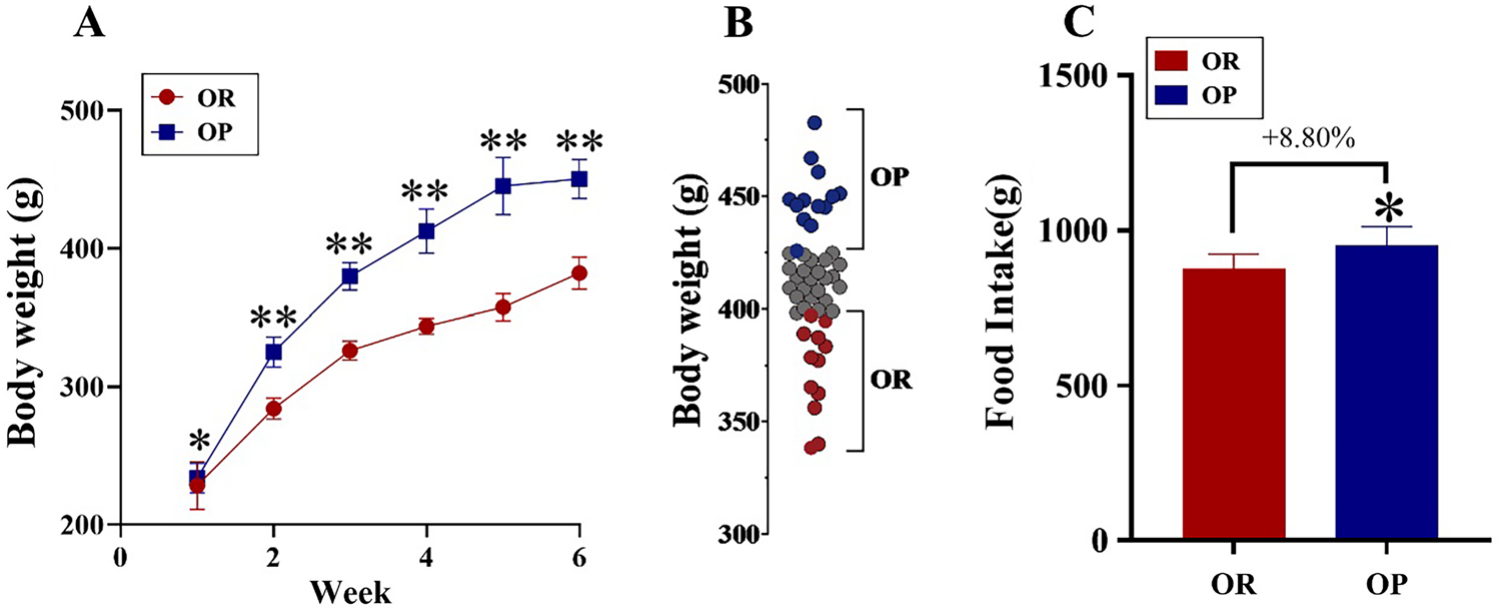
Body weight and food intake during the model establishment period. **A** Body weight changes during the 6-week model establishment period, analysed by repeated measures analysis of variance. **B** Body weight distribution after 6 weeks, with the fourth and first quartiles (n = 12 each) of rats selected as obesity prone (OP) and obesity resistant (OR) rats, respectively. **C** The total food intake during the 6-week model establishment period, analysed by t-test. *P < 0.05, **P < 0.01

During the 6-week model establishment period, body weight increased steadily and was always higher in the OP compared with the OR group (P < 0.05, Fig. 2A). In week 6, OP and OR rats were selected based on their final weight (Fig. 2B). The total food intake of OP rats after 6 weeks was significantly higher than the food intake of OR rats (P < 0.05, Fig. 2C).

### Body weight and food intake during and after 12 weeks of HIIT

**Fig. 3:**
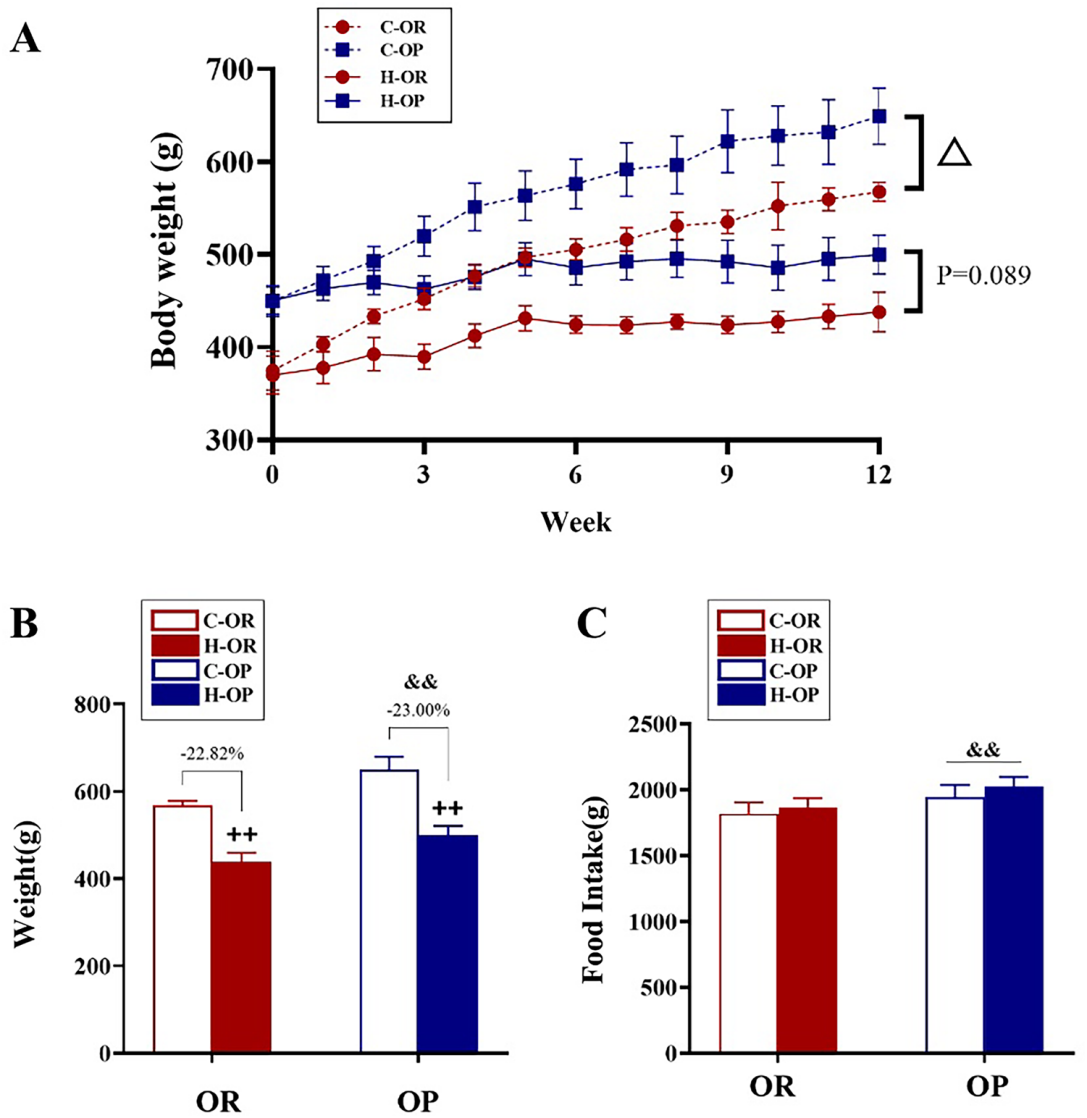
Body weight and food intake during and after 12 weeks of high-intensity interval training (HIIT). **A** Body weight changes during 12 weeks of HIIT, analysed by repeated measures analysis of variance (ANOVA); ^△^time × OP/OR P < 0.05. **B** Body weight at week 12; ^ ++^main effect of HIIT/sedentary P < 0.01, analysed by two-way ANOVA. **C** The total food intake during training; ^&&^main effect of OP/OR P < 0.01, analysed by two-way ANOVA

During the training period, HIIT reduced the body weight in both exercise groups (time × HIIT/sedentary P < 0.01) and the difference between C-OP and C-OR was still increasing (time × C-OP/C-OR P < 0.01, Fig. 3A). HIIT seemed to suppress the increasing trend of body weight in the H-OP relative to the H-OR group (time × H-OP/H-OR P = 0.089, Fig. 3A). However, the data from week 12 showed that HIIT had no effect on reducing the body weight of OP and OR rats (− 23.00% and − 22.82%, respectively, HIIT/Sedentary × OP/OR P > 0.05, Fig. 3B). Regardless of whether they were subjected to HIIT or a sedentary lifestyle, OP rats consumed more food than OR rats (HIIT/Sedentary × OP/OR P > 0.05, main effect of OP/OR P < 0.01, Fig. 3C).

### Fat mass, VAT, mitochondrial histology and TBX1 expression

**Fig. 4:**
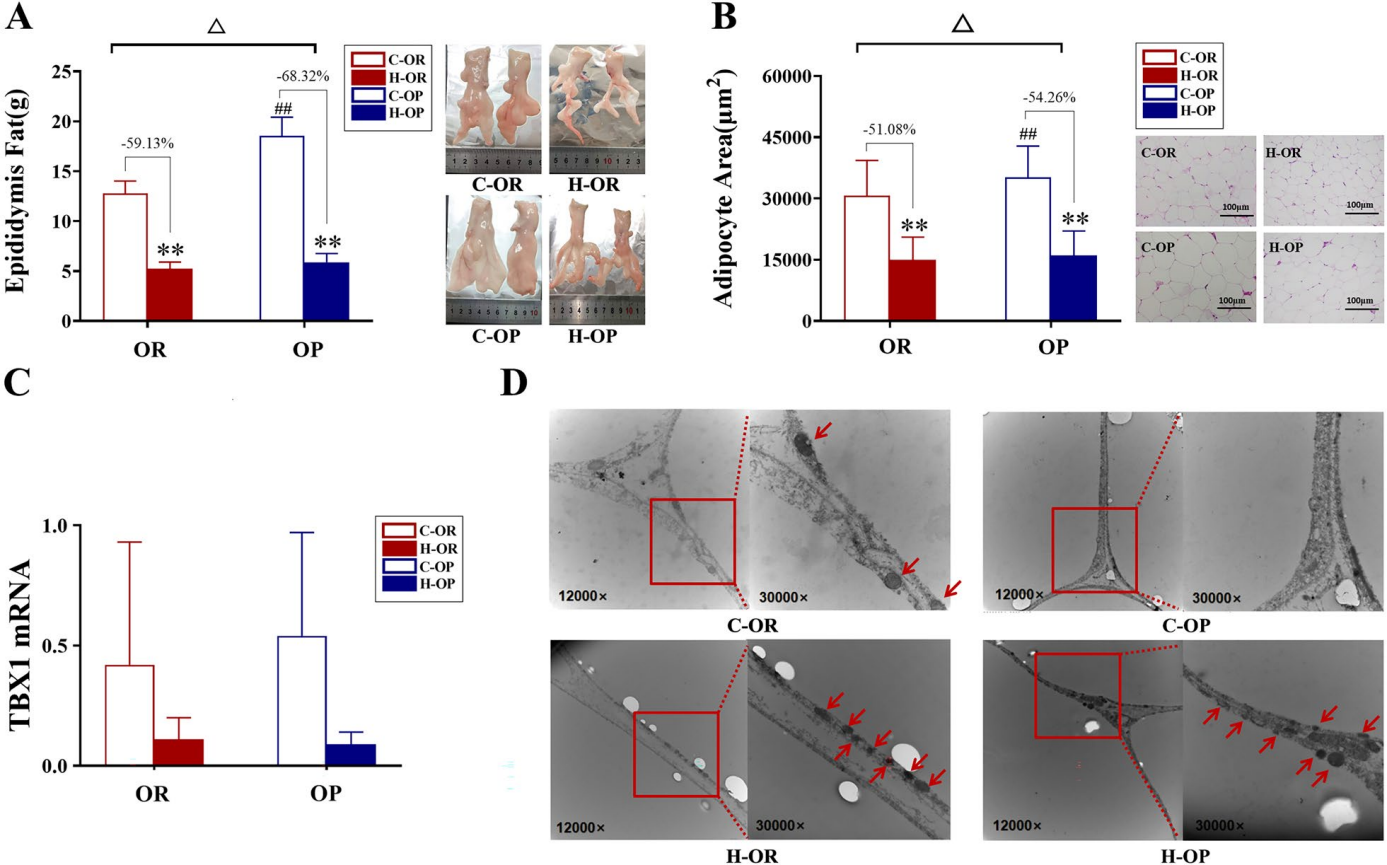
Fat Mass, VAT, TBX1 expression and mitochondrial histology. **A** Epididymal fat mass. **B** Visceral adipocyte area. **C** TBX1 mRNA expression. **D** Transmission electron micrographs showing mitochondria in adipocytes (denoted by red arrows). For **A**, **B** ^△^HIIT/Sedentary × OP/OR P < 0.05. **H-group vs C-group P < 0.01; ^##^C-OP vs C-OR P < 0.01, analysed by simple effect

Data showed that 12 weeks of HIIT could effectively reduce the epididymal fat mass and the visceral adipocyte volume in both the H-OP and H-OR groups (all P < 0.01, Fig. 4A, B) and it should be noted that OP rats showed a greater decline than OR rats (HIIT/Sedentary × OP/OR P < 0.05, Fig. 4A, B). Although due to the large individual differences, there were differences between groups regarding TBX1 mRNA expression, 12 weeks of HIIT increased the number of mitochondria accumulated near lipid droplets in both exercise groups (Fig. 4D). In summary, HIIT showed a stronger effect on reducing VAT in OP rats.

### Blood and liver lipids

**Fig. 5:**
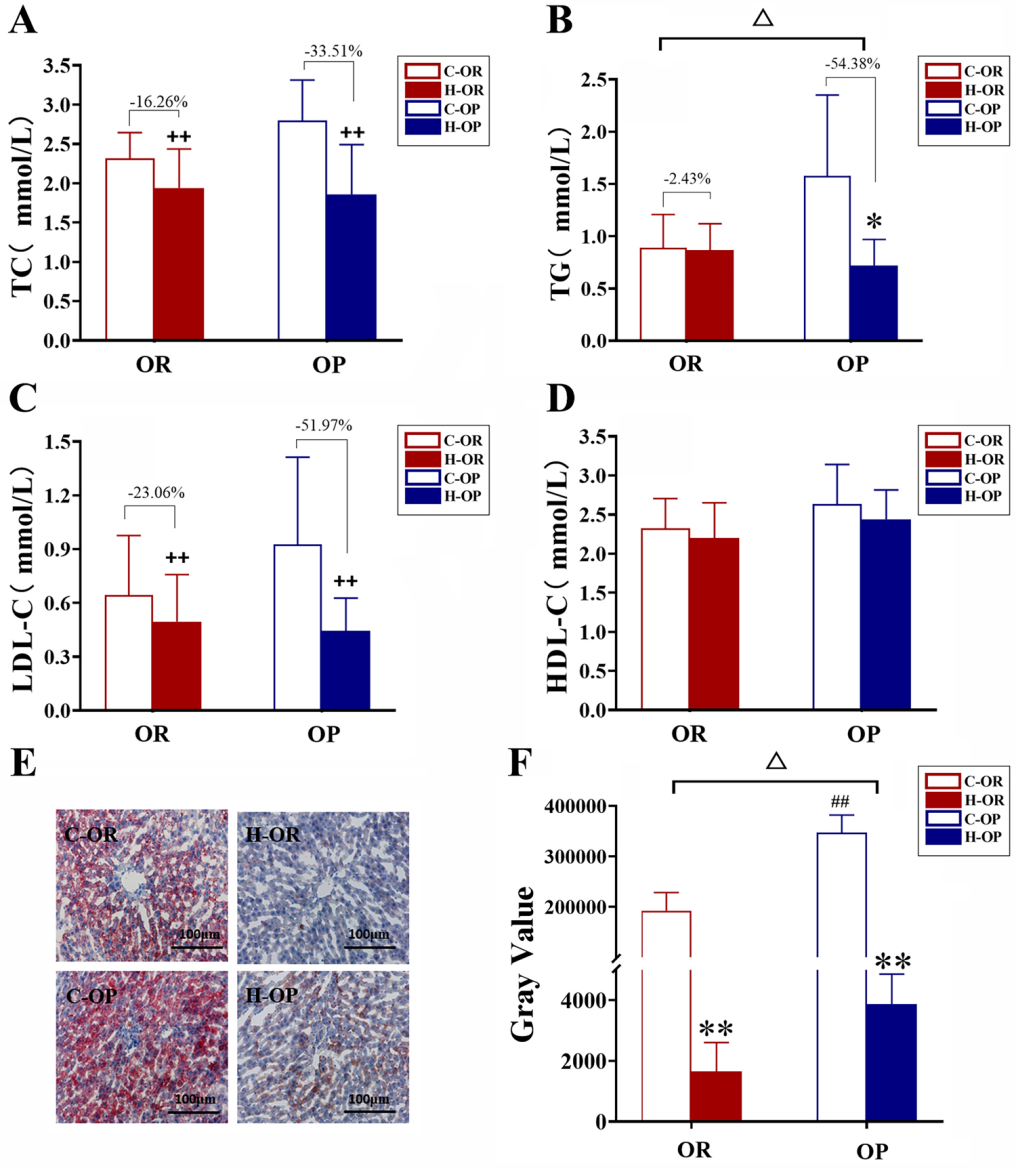
TC, TG, LDL-C, HDL-C and liver histology. **A** Serum total cholesterol. **B** Serum triglycerides. **C** LDL-C. **D** HDL-C. **E** Oil red O staining of liver. **F** Lipid deposition of liver (grey value of Oil red O). For **A**–**D**, **F** △, HIIT/Sedentary × OP/OR P < 0.05, ^ ++^main effect of HIIT/sedentary P < 0.01, analysed by two-way ANOVA.; *H-group vs C-group P < 0.05, **P < 0.01; ^##^C-OP vs C-OR P < 0.01, analysed by simple effect

Data showed that 12 weeks of HIIT could effectively decrease the serum TC, LDL-C and liver lipid deposition in both the H-OP and H-OR groups (all P < 0.01, Fig. 5A, C, F). Only OP rats showed a decline in TG after training (P < 0.05, Fig. 5B). Similar to TG, OP group showed lower liver lipids than OR group (time × H-OP/H-OR P<0.05, Fig. 5F). In summary, HIIT showed a stronger effect on reducing TG and liver lipids in OP rats.

### Expression of TH, β3-AR, HSL and phorylated HSL-ser660 protein

**Fig. 6:**
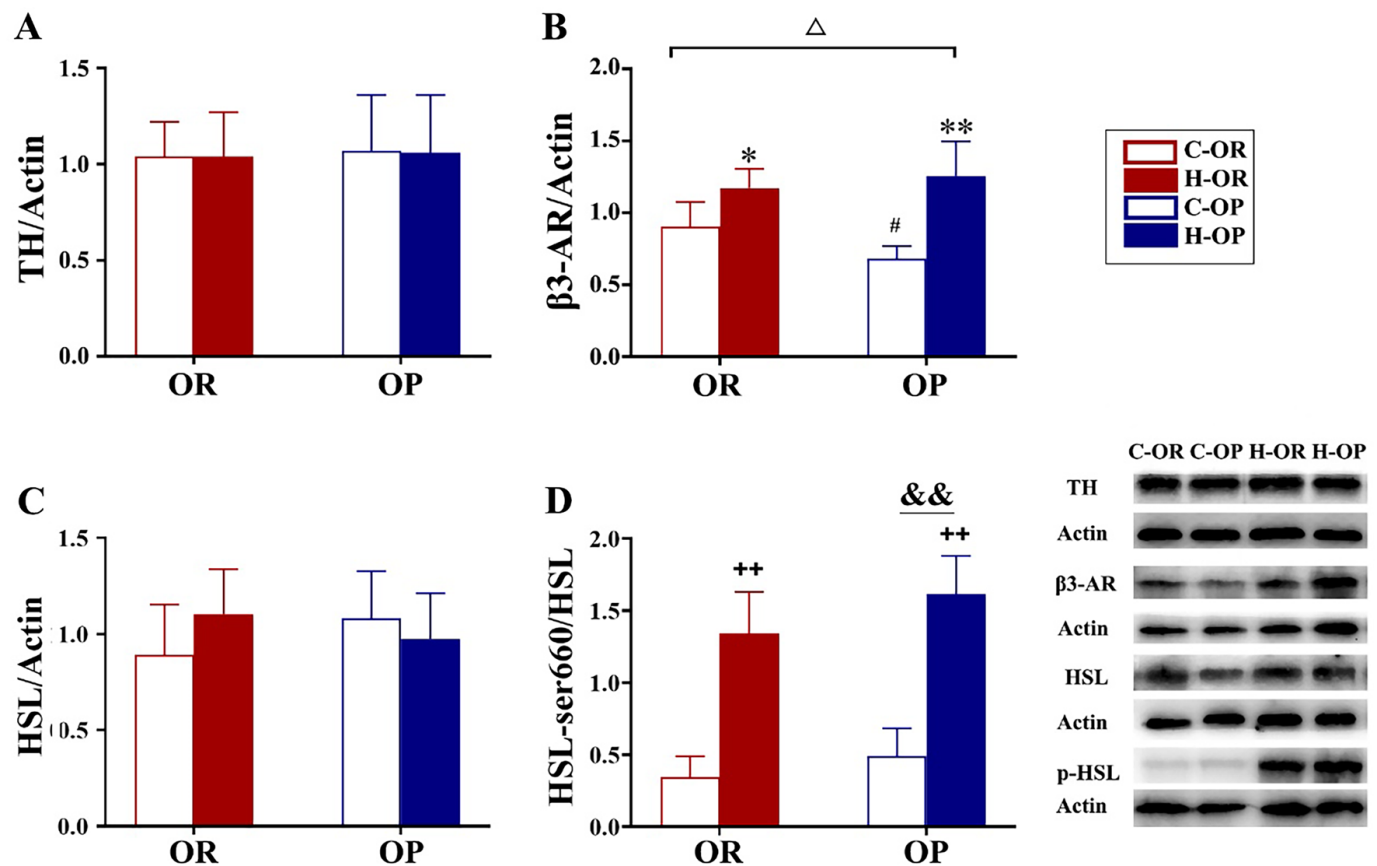
Expression of hormone-sensitive lipase (HSL) and phosphorylated HSL-ser660. **A** TH protein expression. **B** β3-AR protein expression. **C** HSL protein expression. **D** Phosphorylated HSL-ser660 protein expression. ^△^HIIT/Sedentary × OP/OR P < 0.05, analysed by two-way analysis of variance. ^#^C-OP vs C-OR P < 0.05; *H-OR vs C-OR P < 0.05; and **H-OP vs H-OR P < 0.01, analysed by simple effect; ^ ++^main effect of HIIT/sedentary P < 0.01; ^&&^main effect of OP/OR P < 0.01, analysed by two-way analysis of variance

Lipolys is mainly activated by sympathetic nerves that release NE through adrenergic receptors and the cyclic adenosine monophosphate (cAMP)–protein kinase A (PKA) pathway. Hence, we examined the expression of two proteins that are part of the upstream pathway: TH, a marker of NE release, and β3-AR, the major NE receptor of adipocytes as well as HSL (key lipolysis enzyme) total and phorylated protein Expression. TH expression was not affected by the obesity phenotype or training (P > 0.05, Fig. 6A). By contrast, HIIT increased β3-AR expression in both groups (P < 0.05, Fig. 6B). The increase in OP rats was greater than in OR rats (HIIT/Sedentary × OP/OR P < 0.05, Fig. 6B). After 12 weeks of HIIT, neither the obesity phenotype nor training affected HSL expression (P > 0.05, Fig. 6C). OP rats showed higher HSL phosphorylation during starvation than OR rats (P < 0.01, Fig. 4B). These data suggest that when facing fasting pressure, the OP phenotype responds more strongly. HIIT significantly increased phosphorylated HSL-ser660 in all exercise groups (P < 0.01, Fig. 6D), and this training-induced increase was similar regardless of the obesity phenotype (HIIT/Sedentary × OP/OR P > 0.05, Fig. 6D). In summary, these results suggest that OP rats exhibit a stronger adaptive change to HIIT due to increased β3-AR expression rather than increased SNS activity.

### Lipolytic activity of adipocytes induced by in vitro catecholamine stimulation and target β3-AR blockade

**Fig. 7:**
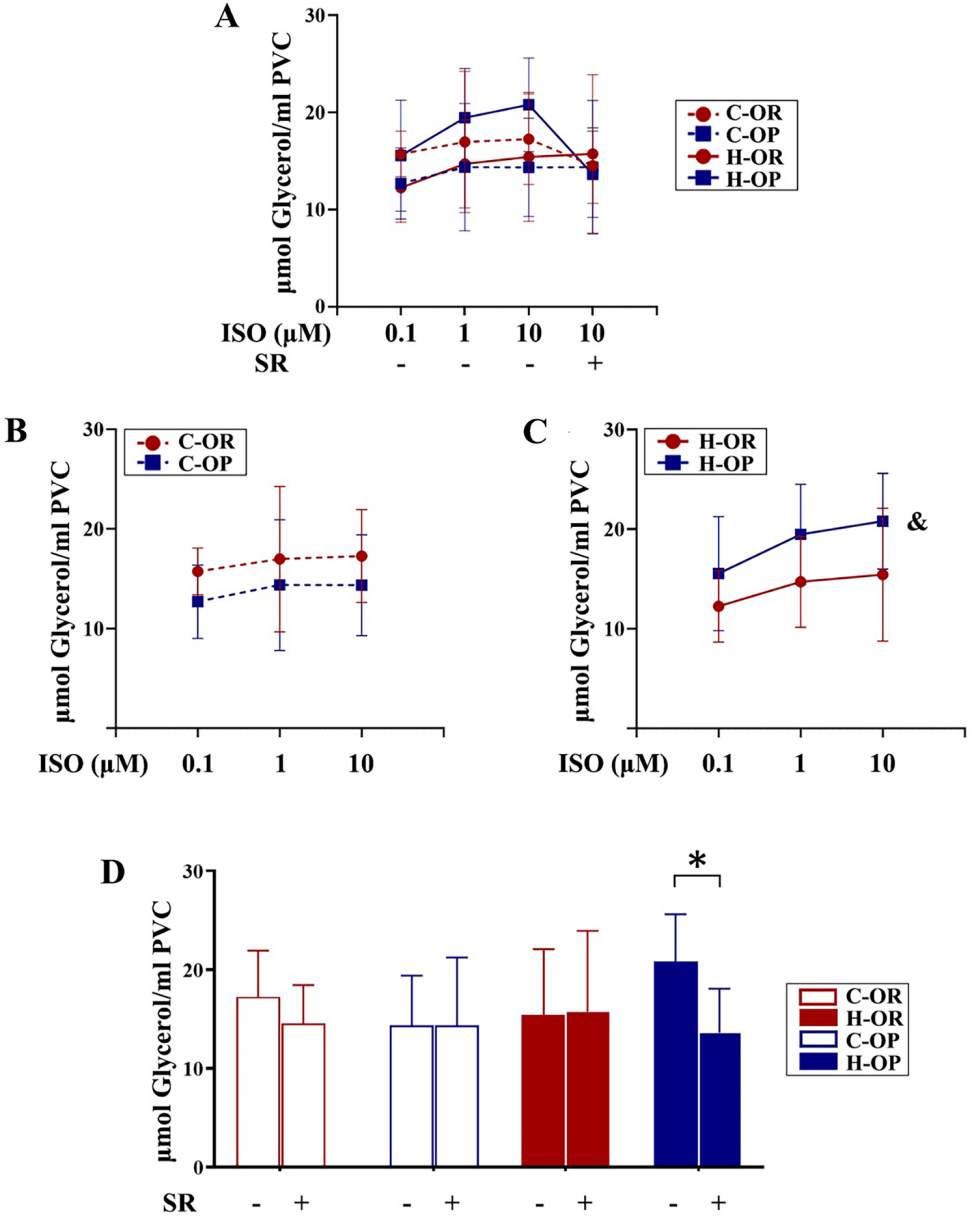
Glycerol release from adipocytes stimulated by isoproterenol (ISO) in vitro. **A** Overview of the glycerol release of all adipocyte groups. **B** Comparison between the control (C) groups. **C** Comparison between the exercise (H) groups; ^&^main effect of OP/OR P < 0.05, analysed by two-way analysis of variance. **D** Changes in glycerol release with or without β3-adrenergic receptor blockade with SR59230a; *P < 0.05, analysed by t-test; *ISO* isoproterenol, *SR* SR59230a

To evaluate whether altered β3-AR expression leads to greater sensitivity to catecholamine, visceral adipocytes of rats submitted to 12 weeks of training and not fasted before sacrifice were isolated and stimulated by an ISO gradient (0.1–10 μM, to imitate hunger) in vitro. Released glycerol (a product of lipolysis) was measured to represent the downstream activation of β3-AR. An overview of each group is presented in Fig. 7A. Glycerol release was similar in the two control groups (P > 0.05, Fig. 7B). However, the lipolysis of the H-OP group was stronger than that of the H-OR group (P < 0.05, Fig. 7C), suggesting that OP rats became more sensitive to catecholamine after training. To further examine whether the elevated sensitivity was related to β3-AR, a selective antagonist (SR59230a) was added to the 10 μM ISO stimulant. With this combination, the increased lipolysis of H-OP was inhibited (P < 0.05, Fig. 7D), indicating that greater β3-AR expression in OP rats plays a key role in mediating the increased catecholamine sensitivity.

## Discussion

HIIT has been confirmed to reduce VAT and relieve metabolic syndrome effectively [1, 5–7]. However, individual differences between subjects are large [1]. A previous meta-analysis found that the effect of HIIT on reducing VAT is evident in subjects with obesity or overweight but weak in normal weight subjects [5]. This phenomenon may be related closely to the difference between obesity phenotypes in adipose catabolism. Previous experiments have found that the fat loss effect of HIIT is related to the activation lipolysis [26–28, 31]. Because gene polymorphisms underlie individual differences in food intake, PA and adipose metabolism, we hypothesised that OP and OR individuals show different adaptive changes to HIIT, especially in lipolysis regulated by the SNS.

We compared how 12 weeks of HIIT affected VAT loss in OP and OR rats, as well as how this training affected catecholamine-regulated lipolysis pathway. We found: (1) HIIT could reduce HFD-induced body weight gain, liver fat and VAT mass in both OP/OR rats, and the influence in OP rats was even stronger. (2) After training, VAT of OP rats was more sensitive to catecholamine and showed stronger lipolysis compared with OR rats. (3) Increased β3-AR expression, rather than increased SNS activity, plays a key role in mediating these adaptive changes.

### HIIT had a stronger influence on body weight, VAT mass, blood and liver lipids in OP compared with OR rats

Similarly to the heterogeneity of human obesity, rodents also show two different obesity phenotypes, namely OP and OR [14, 15]. Although the baseline weights of these rats are the same, marked differences in food intake, PA, central regulation and peripheral tissue metabolism lead OP rats to become obese after several weeks of receiving an HFD, while OR rats tend to remain at a normal weight [32–34]. We established a model of OP and OR rats by using a classic programme involving an HFD [14, 15, 34]. While establishing the model, the food intake and weight gain of OP rats were significantly higher than for OR rats, indicating that the model was established successfully.

Studies have shown that when the environment changes, OR individuals often exhibit a steady state of energy metabolism, while OP individuals respond more strongly to caloric restriction or food surplus [35, 36], suggesting that the OP phenotype has a survival advantage when energy is insufficient but is harmful during periods of excess food. Previous comparative studies of OP and OR have often focussed on aerobic exercise [20–22, 35, 36]. However, differently from aerobic exercise, HIIT consumes glycogen but not fat during exercise. There is an observation that many effective treatments (intermittent fasting, caloric restriction, pharmacological treatments, etc.) for metabolic syndrome have in common the ability to decrease liver and muscle glycogen and increase ketone bodies [37, 38]. Theoretically, HIIT has a similar glycogen-depleting effect, and OP phenotype generally shows faster fat synthesis/decomposition alternations. After HIIT, more intense lipolysis may occur in VAT of OP individuals, which could meet liver fatty acid and glycerol demands for ketogenisis and gluconeogenesis. However, whether HIIT could reduce VAT in OP phenotype more efficiently requires new evidence.

To our knowledge, this study compared the fat-loss effects of HIIT on OP and OR subjects for the first time. We found that HIIT had a stronger effect on reducing VAT in OP than OR rats, indicating that the differential adaptation of OP and OR subjects may be the reason for the strong heterogeneity of the HIIT fat-loss effect. Because the 12 weeks of training had no effect on the food intake, the heterogeneity of the VAT decrease between OP and OR rats may be more related to altered peripheral metabolism than feeding regulation by the central nervous system. The VAT mass in adulthood is more related to the volume rather than the number of adipocytes, and the main contributor (> 95%) of the cell volume comes from TG accumulation in lipid droplets [39, 40]. We found that after 6 weeks of an HFD, the adipocyte areas of OP rats were larger than that of OR rats, a finding that is characteristic of the classic OP animal model [41, 42]. After 12 weeks of HIIT, the cell area had decreased in both OP and OR rats, although the reduction was greater in OP rats, accompanied by an increase in the number of mitochondria (mean higher local lipid oxidation), suggesting that HIIT reduces the TG content of VAT in OP rats more strongly than OR.

Central obesity is a key component of metabolic syndrome. Excessive VAT accumulation is associated with hyperlipidaemia and non-alcoholic fatty liver disease (NAFLD) [43]. Exercise-induced decreases in VAT are often accompanied by decreased blood and liver lipids [44]. Our results are consistent with previous findings: the decreases of serum TG and liver lipids in OP rats were larger than in OR rats, indicating greater health benefits in the former group.

Generally, adipocytes of OP phenotype are more likely to enhance fat uptake after overfeeding [36]. Excessive VAT accumulation in could cause hypoxia, inflammation and IR, and these state will induce HSL activation and lipid overflow through the portal vein to liver and muscle (hyperlipidaemia and ectopic fat deposition) [38], greatly increasing the risk of NAFLD. As described above, HIIT may cause the exhaustion of glycogen and non-pathological ketogenesis, which have been shown to improve obesity and dysfunctional glucose/lipid metabolism [24]. After each session of HIIT, the adaptive dynamic rising of lipolysis in VAT may emerge for ketogenesis and gluconeogenesis in liver. This process is different from ‘uncontrolled’ lipolysis and lipid overflow and could be a healthy adaptation. In summary, HIIT showed a greater influence on the VAT mass, cell volume, hyperlipidaemia and liver lipid deposition of OP compared with OR rats, findings that imply a stronger catabolic change in adipose tissue of OP individuals.

### VAT of OP rats showed greater lipolytic potential than OR rats after HIIT

Based on the data that the VAT of OP rats decreased more than OR rats after training, we hypothesised that heterogeneity in VAT changes between OP and OR rats is related to the different adaptations of adipocyte lipolytic pathways. Lipolysis is the primary step of TG decomposition, which refers to the process by which TG are hydrolysed into glycerol and non-esterified fatty acids (NEFA, the released form of TG). This process is mainly regulated by AR in the SNS [45, 46]. The SNS releases NE and epinephrine through nerve endings and adrenal glands, which can activate second messenger pathway (involving G proteins and cAMP) through AR, ultimately leading to PKA-mediated phosphorylation of HSL and activation of lipolysis [47]. Catecholamine release is correlated with exercise intensity [48]. Aerobic exercise mediates moderate secretion, which enhances lipolysis through β-AR of adipocytes to meet fat consumption during exercise, while high-intensity exercise induces excessive secretion of catecholamine and inhibits lipolysis through a negative feedback mechanism involving α-AR [49, 50].

Because minimal fat is burned during exercise, it is generally believed that HIIT can reduce fat based on post-exercise TG consumption [23, 24, 51]. A commonly mentioned view is that HIIT could increase excess post-exercise oxygen consumption (EPOC), but there are still controversies among existing results [52]. Another reasonable hypothesis is that due to the intense load of HIIT, during the recovery period, adipose tissue would release more lipid for gluconeogenesis, ketogenesis or tissue healing [24], which suggests that HIIT promotes adaptive catabolism of adipose tissue. Studies have verified the increase in β-AR and lipolysis in adipocytes after long-term HIIT [26, 28], supporting the hypothesis of adaptive changes in SNS lipolytic pathway. As mentioned earlier, when facing catabolic stress, OP individuals are more likely to show adaptation (fat is lost easily), while OR individuals are more likely to maintain homeostasis [35, 36]. Therefore, a reasonable explanation for the greater decrease in VAT mass of OP rats than OR ones after HIIT is that the visceral adipocytes of the former group generate a stronger adaptation to HIIT. SNS lipolytic pathway of OP phenotype is easier to start-up in face of gluconeogenesis, ketogenesis, tissue healing or metabolic stress.

It should be noted that excessive accumulation of VAT could result in IR and inhibition of the insulin receptor-PI3K/Akt pathway in adipocytes, which can also cause ‘uncontrollable’ HSL phosphorylation and lipolysis activation. Unlike IR-induced lipolysis, the SNS lipolytic pathway is only activated when the lipid demand of other organs increases. This process could be a healthy adaptation that does not induce hyperlipidaemia and ectopic fat deposits [44, 53]. Therefore, we hypothesized that HIIT increased VAT lipolysis via a mechanism related to adaptive changes in SNS-AR pathway but not the insulin receptor pathway.

To verify this hypothesis, we evaluated the expression and phosphorylation of HSL as well as catecholamine-induced glycerol release (lipolysis marker) from adipocytes in vitro. We found that after 12 h of fasting (inducing catecholamine release), there was no difference in HSL expression between the groups, but the level of phosphorylated HSLser660 was higher in the exercise groups than in the control groups, indicating an activating effect of HIIT on VAT lipolysis. Our in vitro experiment confirmed higher rate of lipolysis in the H-OP group after catecholamine stimulation, suggesting that there was stronger catabolism in the OP rats. In summary, these results suggest greater adaptation of the lipolysis regulation pathway in OP compared with OR rats.

### Increased β3-AR expression may play a key role in mediating adaptive change to HIIT

Although VAT lipolysis in the H-OP group showed stronger adaptations to HIIT, the upstream mechanisms that mediate these changes remained unknown. Adipose tissue is regulated extensively by the neuroendocrine network. Although the SNS plays a primary role [54], parasympathetic nerves, natriuretic peptides, glucocorticoids and parathyroid hormone are also involved in regulating lipolysis [55]. Even downstream of the SNS, catecholamine and AR are not the only ‘transmitter–receptor’ pathway. Recent studies have reported other transmitters, receptors or intermediary cells such as neuropeptide Y (NPY), G-protein coupled receptor 3 (GPR3) and adipose mesenchymal cells (MSCs) involved in the SNS-mediated regulation of adipose catabolism [56–58]. Due to the existence of many pathways, the role of the SNS and β3-AR needed to be further confirmed in this study.

Because the hypothalamus is the major regulator of energy homeostasis and directly controls the SNS and food intake [54, 59], we speculated that the hypothalamus of OP rats could adapt to HIIT, thereby changing the feeding behaviour and SNS-regulated adipose metabolism. Unexpectedly, similarly to the food intake, results of TH expression didn’t support that HIIT promote NE release of sympathetic nerves. Whether changes in activity of the hypothalamus and SNS were related to the reduction of VAT required more experiments to explore. However, β3-AR expression of the exercise groups was increased significantly, and the increase was greater in the H-OP compared with the H-OR group, indicating that HIIT could increase more strongly the receptor number and the exercise adaptation of OP rats. To determine whether higher β3-AR expression produced stronger sensitivity to catecholamine, isolated adipocytes were stimulated by an ISO gradient (0.1–10 μM, to imitate levels of catabolism pressure) in vitro. The glycerol release of the H-OP group was significantly higher than the H-OR group, suggesting that adipocytes of OP rats are more sensitive to catecholamine and could produce more NEFA to export or local oxidation under the same SNS signal.

So far, three beta isoforms of AR have been reported (β1–β3). Although β3-AR predominates in white adipose tissue [60], studies have confirmed that gene polymorphisms of all three isoforms could affect the fat catabolism induced by aerobic exercise [61–63], although the relationship between HIIT and different isoforms had been unclear. To determine whether the VAT adaptation to HIIT is only related to β3-AR or also to other isoforms, we blocked this receptor with SR59230a, a selective antagonist, concomitantly with 10 μM ISO stimulation. Without the participation of β3-AR, the increased glycerol release in the H-OP group disappeared. These data confirm that increased expression of β3-AR, rather than β1-AR or β2-AR, plays a key role in increasing the sensitivity of adipocytes to catecholamine. In summary, after 12 weeks of HIIT, greater β3-AR expression in OP rats led to an elevated sensitivity to catecholamine compared with the OR rats. This change underlies the greater VAT lipolysis capabilities of OP rats.

## Limitations

Our study had some limitations. First, due to species differences of β-AR, additional studies are needed to confirm whether the conclusions of this study are applicable to humans. Second, because of equipment limitations, basal or fasting metabolic monitoring as well as body fat percentage measurement was not carried out on rats. Third, additional assessments of the hypothalamus and SNS, such as neuron function, traffic, transmitter release, etc. are needed to establish a more general neurohormonal basis for HIIT-related VAT control.

## Conclusion

HIIT provides notable advantages in reducing metabolic syndrome manifestations and is more time-efficient than aerobic exercise, but the fat-loss effects in different populations are highly heterogeneous, and the reason may be related to difference in adaptive changes in VAT lipolysis between obesity phenotypes. We found that 12 weeks of HIIT reduced visceral fat more in OP compared with OR rats. After training, the VAT lipolytic capabilities of OP rats were higher than those of OR rats, indicating that the OP phenotype shows greater catabolic adaptation to HIIT. In addition, increased β3-AR expression played a key role in mediating this process. Future research is needed to verify whether HIIT-induced VAT reduction, along with blood and liver lipids improvement, could improve the IR of adipocytes and ectopic fat deposition in other organs, an endeavour that could help to develop more effective methods for controlling the risk of central obesity.

## Data Availability

The datasets used and analyzed during the current study are available from the corresponding author on reasonable request.

## Abbreviations

HIIT: High-intensity interval training
OP: Obesity prone
OR: Obesity resistant
β3-AR: β3-Adrenergic receptor
VAT: Visceral adipose tissue
SNS: Sympathetic nervous system
HFD: High-fat diet
NE: Norepinephrine
TG: Triglyceride
TC: Total cholesterol
LDL-C: Low-density lipoprotein cholesterol
HDL-C: High-density lipoprotein cholesterol
TH: Tyrosine hydroxylase
HSL: Hormone-sensitive lipase
ISO: Isoproterenol
EPOC: Post-exercise oxygen consumption
